# Multivariate Analysis under Indeterminacy: An Application to Chemical Content Data

**DOI:** 10.1155/2020/1406028

**Published:** 2020-07-11

**Authors:** Muhammad Aslam, Osama H. Arif

**Affiliations:** ^1^Department of Statistics, Faculty of Science, King Abdulaziz University, Jeddah 21551, Saudi Arabia; ^2^Department of Mathematics, Faculty of Science, Jouf University, Sakakah, Saudi Arabia

## Abstract

The Hotelling T-squared statistic has been widely used for the testing of differences in means for the multivariate data. The existing statistic under classical statistics is applied when observations in multivariate data are determined, precise, and exact. In practice, it is not necessary that all observations in the data are determined and precise due to measurement in complex situations and under uncertainty environment. In this paper, we will introduce the Hotelling T-squared statistic under neutrosophic statistics (NS) which is the generalization of classical statistics and applied under uncertainty environment. We will discuss the application and advantage of the neutrosophic Hotelling T-squared statistic with the aid of data. From the comparison, we will conclude that the proposed statistic is more adequate and effective in uncertainty.

## 1. Introduction

In classical statistics (CS), the univariate analysis is the technique to analyze the single-variable data. The multivariate analysis has been widely used to analyze data having more than one variable. In the multivariate technique under the CS, the Hotelling T-squared statistic has been widely applied in the variety of fields (see, for example, [1, 2]), for the testing either the means for more than one populations are equal or not. This statistic is the extension of the *t*-test, which is applied for the testing of the mean for the single population. Brereton [3] used the Hotelling T-squared statistic to detect the outlier in chemical data. In [4], Varmuza and Filzmoser worked on multivariate analysis for chemometric data. Hervé et al. [5] applied the multivariate technique on biological data. Kitaga ki et al. [6] used Hotelling T-squared statistic in chemical and electrochemical oscillator issues. For more details about the applications of the Hotelling T-squared statistic, the reader may read [3, 7] and [8].

The Hotelling T-squared statistic derived under the CS can be only applied for the analysis when all observations in the multivariate data are determined, precise, and certain. In practice, the data under study are not always precise but linguistic. For example, the temperature of a certain city may be high, low, and medium or the measurement of variable data in a complex system may lead to being in an interval rather than the determined values. In such situations, the Hotelling T-squared statistic under the CS cannot be used for the analysis of the data. When observations are uncertain or fuzzy, the fuzzy Hotelling T-squared statistic can be applied for the testing of means of multivariate populations. Taleb et al. [9] applied the fuzzy Hotelling T-squared statistic to design a control chart. D'Urso [10] provided a review on fuzzy multivariate analysis. Bakdi and Kouadri [11] presented a new adoptive principle component analysis technique to detect fault in a complex system. In [12], Ammiche et al. introduced principle component analysis for the Tennessee Eastman process using a fuzzy approach. More applications can be read in [13–15].

Recently, the neutrosophic logic, which is the extension of the fuzzy logic, attracted many researchers due to its applications in the variety of fields. The neutrosophic logic considered the measure of indeterminacy which fuzzy logic does not consider (see [16]). The neutrosophic statistics (NS) which is based on the neutrosophic numbers is the generalization of the CS (see [17, 18]). The NS has been applied widely in the rock-measuring issues (see, for example, [19, 20]). The application of the NS for the inspection of the product can be seen in [21, 22]. The applications of the NS in the area of the process control can be seen in [23, 24]. The application of the NS in medical can be read in [25]. For more information on neutrosophic theory, the reader may refer to [26, 27].

Aslam and Smarandache [17, 18] pointed out some suggestions to extend the several concepts of CS to the NS. By exploring the literature and best of our knowledge, there is no work on the development of Hotelling T-squared statistic under the NS. In this paper, we will introduce the Hotelling T-squared statistic under NS, which is the generalization of classical statistics and applied under uncertainty environment. We will discuss the application and advantage of neutrosophic Hotelling T-squared statistic with the aid of data. We expect that the proposed neutrosophic Hotelling T-squared statistic will perform better than the existing Hotelling T-squared statistic in uncertainty.

## 2. Preliminaries

Let *x*_*jkN*_*∈*[*x*_*jkL*_, *x*_*jkU*_] be a neutrosophic random variable, which represents the particular neutrosophic observation of the *k*^th^ variable that is noted from the *j*^th^ item. Note here that *x*_*jkN*_*∈*[*x*_*jkL*_, *x*_*jkU*_] is expressed in the indeterminacy interval having the smaller value *x*_*jkL*_and the larger value *x*_*jkU*_. The neutrosophic form of *x*_*jkN*_*∈*[*x*_*jkL*_, *x*_*jkU*_] having determinate part *x*_*jkL*_ and indeterminate part *x*_*jkU*_*I*_*N*_; *I*_*N*_*∈*[*I*_*L*_, *I*_*U*_] can be written as follows: *x*_*jkN*_=*x*_*jkL*_+*x*_*jkU*_*I*_*N*_; *I*_*N*_*∈*[*I*_*L*_, *I*_*U*_]. Note here that the neutrosophic random variable reduces to the variable under classical statistics if no indeterminacy is recorded in the data. The neutrosophic data matrix having *n*_*N*_*∈*[*n*_*L*_, *n*_*U*_] neutrosophic observations of *p*_*N*_*∈*[*p*_*L*_, *p*_*U*_] neutrosophic variables is given as follows:

(1) $$\begin{array}{c} X_{N}\epsilon \left[\left[\begin{array}{ccc} x_{11L}\cdots & x_{1kL}\cdots & x_{1pL}\\ x_{j1L}\cdots & x_{j1L}\cdots & x_{jpL}\\ x_{n1L}\cdots & x_{nkL}\cdots & x_{npL} \end{array}\right],\left[\begin{array}{ccc} x_{11U}\cdots & x_{1kU}\cdots & x_{1pU}\\ x_{j1U}\cdots & x_{j1U}\cdots & x_{jpU}\\ x_{n1U}\cdots & x_{nkU}\cdots & x_{npU} \end{array}\right]\right];X_{N}\epsilon \left[X_{L},X_{U}\right]. \end{array}$$

The neutrosophic form of *X*_*N*_*ϵ*[*X*_*L*_, *X*_*U*_] can be written as

(2) $$\begin{array}{c} X_{N}=X_{L}+X_{U}I_{N};I_{N}\epsilon \left[I_{L},I_{U}\right]. \end{array}$$

Note here that *X*_*N*_*ϵ*[*X*_*L*_, *X*_*U*_] is the generalization of the data matrix under classical statistics. The data matrix under *X*_*N*_*ϵ*[*X*_*L*_, *X*_*U*_] reduces to the data matrix under classical statistics when *I*_*L*_ = 0.

The neutrosophic sample mean and neutrosophic sample variance from *n*_*N*_ measurements from *p*_*N*_ neutrosophic variables are computed as follows:

(3) $$\begin{array}{c} {\bar{x}}_{kN}\epsilon \left[\left[\frac{1}{n_{L}}\sum_{j=1}^{n_{L}}x_{jkL}\right],\left[\frac{1}{n_{U}}\sum_{j=1}^{n_{U}}x_{jkU}\right]\right];{\bar{x}}_{kN}\epsilon \left[{\bar{x}}_{kL},{\bar{x}}_{kU}\right]. \end{array}$$

The neutrosophic form of ${\bar{x}}_{kN}\epsilon \left[{\bar{x}}_{kL},{\bar{x}}_{kU}\right]$ can be written as

(4) $$\begin{array}{c} {\bar{x}}_{kN}={\bar{x}}_{kL}+{\bar{x}}_{kU}I_{N};I_{N}\epsilon \left[I_{L},I_{U}\right]. \end{array}$$

Note here that ${\bar{x}}_{kN}\epsilon \left[{\bar{x}}_{kL},{\bar{x}}_{kU}\right]$ is the generalization of the sample mean under classical statistics. The data matrix under ${\bar{x}}_{kN}\epsilon \left[{\bar{x}}_{kL},{\bar{x}}_{kU}\right]$ reduces to the sample mean under classical statistics when *I*_*L*_ = 0:

(5) $$\begin{array}{c} s_{kN}^{2}\epsilon \left[\left[\frac{1}{n_{L}}\sum_{j=1}^{n_{L}}{\left(x_{jkL}-{\bar{x}}_{kL}\right)}^{2}\right],\left[\frac{1}{n_{U}}\sum_{j=1}^{n_{U}}{\left(x_{jkU}-{\bar{x}}_{kU}\right)}^{2}\right]\right];s_{kN}^{2}\epsilon \left[s_{kL}^{2},s_{kU}^{2}\right]. \end{array}$$

The neutrosophic form of *s*_*kN*_^2^*ϵ*[*s*_*kL*_^2^, *s*_*kU*_^2^] can be written as

(6) $$\begin{array}{c} s_{kN}^{2}=s_{kL}^{2}+s_{kU}^{2}I_{N};I_{N}\epsilon \left[I_{L},I_{U}\right]. \end{array}$$

Note here that *s*_*kN*_^2^*ϵ*[*s*_*kL*_^2^, *s*_*kU*_^2^] is the generalization of sample variance under classical statistics. The data matrix under *s*_*kN*_^2^*ϵ*[*s*_*kL*_^2^, *s*_*kU*_^2^] reduces to the sample variance under classical statistics when *I*_*L*_ = 0.

The neutrosophic sample covariance between two neutrosophic variables are given by

(7) $$\begin{array}{c} S_{ikN}\epsilon \left[\left[\frac{1}{n_{L}}\sum_{j=1}^{n_{L}}\left(x_{jiL}-{\bar{x}}_{kL}\left(\left(x_{jkL}-{\bar{x}}_{kL}\right)\right)\right)\right],\left[\frac{1}{n_{U}}\sum_{j=1}^{n_{U}}\left(x_{jiU}-{\bar{x}}_{kU}\left(\left(x_{jkU}-{\bar{x}}_{kU}\right)\right)\right)\right]\right];S_{ikN}\epsilon \left[S_{ikL},S_{ikU}\right]. \end{array}$$

The neutrosophic form of *S*_*ikN*_*ϵ*[*S*_*ikL*_, *S*_*ikU*_] can be written as

(8) $$\begin{array}{c} S_{ikN}=S_{ikL}+S_{ikU}I_{N};I_{N}\epsilon \left[I_{L},I_{U}\right]. \end{array}$$

Note here that *S*_*ikN*_*ϵ*[*S*_*ikL*_, *S*_*ikU*_] is the generalization of sample covariance under classical statistics. The data matrix under *S*_*ikN*_*ϵ*[*S*_*ikL*_, *S*_*ikU*_] reduces to the sample covariance under classical statistics when no indeterminate observations.

Finally, neutrosophic sample correlation between the *i*^th^ and *k*^th^ variables is given by

(9) $$\begin{array}{c} r_{ikN}\epsilon \left[\left[\frac{S_{ikL}}{\sqrt{s_{iiL}}\sqrt{s_{kkL}}}\right],\left[\frac{S_{ikU}}{\sqrt{s_{iiU}}\sqrt{s_{kkU}}}\right]\right];r_{ikN}\epsilon \left[r_{ikL},r_{ikU}\right],\;i,k=1,2,3,\ldots ,p_{N}. \end{array}$$

The neutrosophic form of *r*_*ikN*_*ϵ*[*r*_*ikL*_, *r*_*ikU*_] can be written as

(10) $$\begin{array}{c} r_{ikN}=r_{ikL},r_{ikU}I_{N};I_{N}\epsilon \left[I_{L},I_{U}\right]. \end{array}$$

Note here that *r*_*ikN*_*ϵ*[*r*_*ikL*_, *r*_*ikU*_] is the generalization of sample correlation under classical statistics. The data matrix under *r*_*ikN*_*ϵ*[*r*_*ikL*_, *r*_*ikU*_] reduces to the sample correlation under classical statistics when no indeterminate observations.

The neutrosophic descriptive statistics for *n*_*N*_ measurements and on *p*_*N*_ variables can be presented into the following arrays.

The neutrosophic sample mean variance and covariance and correlation are presented by the array X¯Nϵx¯1L⋮x¯pL,x¯1U⋮x¯pU,SNϵs11L⋯s1kL⋯s1pLsj1L⋯sj1L⋯sjpLsn1L⋯snkL⋯snpL,s11U⋯s1kU⋯s1pUsj1U⋯sj1U⋯sjpUsn1U⋯snkU⋯snpU andRNϵ1⋯r12L⋯r1pLr21L⋯1⋯s2pLspL1L⋯spL2L⋯1,1⋯r12U⋯r1pUr21U⋯1⋯s2pUspU1U⋯spU2U⋯1,respectively.

## 3. Neutrosophic Hotelling *T*_*N*_^2^ Statistic

In this section, we discuss the proposed neutrosophic Hotelling *T*_*N*_^2^ statistic. In classical statistics, the student *t*-test is applied for the testing of the mean for the univariate case. As mentioned by [28], rejecting the null hypothesis that means are equal when |*t*_*N*_|is large is the same as rejecting the null hypothesis of its square:

(11) $$\begin{array}{c} t_{N}^{2}=\left[\left(\frac{{\left({\bar{X}}_{L}-\mu_{0L}\right)}^{2}}{s_{L}^{2}/n_{L}}\right),\left(\frac{{\left({\bar{X}}_{U}-\mu_{0U}\right)}^{2}}{s_{U}^{2}/n_{U}}\right)\right]\\ =\left[n_{L}{\left({\bar{X}}_{L}-\mu_{0L}\right)}^{2}{\left(s_{L}^{2}\right)}^{-1},n_{U}{\left({\bar{X}}_{U}-\mu_{0U}\right)}^{2}{\left(s_{U}^{2}\right)}^{-1}{\left({\bar{X}}_{U}-\mu_{0U}\right)}^{2}\right];t_{N}^{2}\epsilon \left[t_{L}^{2},t_{U}^{2}\right]. \end{array}$$

The neutrosophic form of *t*_*N*_^2^*ϵ*[*t*_*L*_^2^, *t*_*U*_^2^] can be written as

(12) $$\begin{array}{c} t_{N}^{2}=t_{L}^{2},t_{U}^{2}I_{N};I_{N}\epsilon \left[I_{L},I_{U}\right]. \end{array}$$

Note here that *t*_*N*_^2^*ϵ*[*t*_*L*_^2^, *t*_*U*_^2^] is the generalization of Hotelling T-squared statistic under classical statistics. The data matrix under *t*_*N*_^2^*ϵ*[*t*_*L*_^2^, *t*_*U*_^2^] reduces to the Hotelling T-squared statistic under classical statistics when no indeterminate observations.

For the given values of ${\bar{x}}_{kN}\epsilon \left[{\bar{x}}_{kL},{\bar{x}}_{kU}\right]$ and *S*_*kN*_^2^*ϵ*[*S*_*kL*_^2^, *S*_*kU*_^2^], the null hypothesis will be rejected if

(13) $$\begin{array}{c} t_{N}^{2}\epsilon \left[t_{L}^{2},t_{U}^{2}\right]>t_{n_{U}-1}^{2}\left(\frac{\alpha }{2}\right), \end{array}$$

where *α* is the level of significance and *t*_*n*_*U*_−1_(*α*/2) is upper 100(*α*/2)^th^ percentiles of the neutrosophic *t*-distribution with the neutrosophic degree of freedom *n*_*U*_ − 1.

The generalization of equations (1) and (2) for the multivariate case under the neutrosophic statistical interval method (NSIM) is given by

(14) $$\begin{array}{c} T_{N}^{2}=\left[{\left({\bar{X}}_{L}-\mu_{0L}\right)}^{\prime }{\left(\frac{1}{n_{L}}S_{L}\right)}^{-1}\right]\left({\bar{X}}_{L}-\mu_{0L}\right),{\left({\bar{X}}_{U}-\mu_{0U}\right)}^{\prime }{\left(\frac{1}{n_{U}}S_{U}\right)}^{-1}\left({\bar{X}}_{U}-\mu_{0U}\right);T_{N}^{2}\;\in \left[T_{L}^{2},T_{U}^{2}\right], \end{array}$$

where $\begin{array}{c} {\bar{X}}_{\left(N_{p_{N}}\times 1\right)}=\left(1/n_{N}\right)\sum_{j=1}^{n_{N}}X_{jN},S_{\left(N_{P_{N}}\times_{p_{N}}\right)}=\left(1/n_{N}-1\right){\sum_{j=1}^{n_{N}}\left(X_{jN}-{\bar{X}}_{N}\right)}^{\prime },\mathrm{and}\\ \mu_{0N\left(p_{N}\times 1\right)}\epsilon \left(\left[\begin{array}{c} \mu_{1N}\\ \vdots \\ \mu_{p_{N}} \end{array}\right]\right);{\bar{X}}_{\left(N_{p_{N}}\times 1\right)}\epsilon \left[{\bar{X}}_{\left(L_{p_{N}}\times 1\right)},{\bar{X}}_{\left(U_{p_{N}}\times 1\right)}\right],S_{\left(N_{p_{N}}\times p_{N}\right)}\epsilon \left[S_{\left(L_{p_{N}}\times p_{N}\right)},S_{\left(U_{p_{N}}\times p_{N}\right)}\right],\\ \;\mu_{0N}\epsilon \left[\mu_{0L\left(p_{N}\times 1\right)},\mu_{0U\left(p_{N}\times 1\right)}\right]. \end{array}$

The neutrosophic form of *T*_*N*_^2^*ϵ*[*T*_*L*_^2^, *T*_*U*_^2^] can be written as

(15) $$\begin{array}{c} T_{N}^{2}=T_{L}^{2}+T_{U}^{2}I_{N};I_{N}\epsilon \left[I_{L},I_{U}\right]. \end{array}$$

The statistic is given in equation (14) is called neutrosophic Hotelling *T*_*N*_^2^ statistic and has neutrosophic *F*-distribution with neutrosophic degree of freedom (ndf) *p*_*N*_ and (*n*_*N*_ − *p*_*N*_):

(16) $$\begin{array}{c} T_{N}^{2}∼\frac{\left(n_{N}-1\right)}{\left(n_{N}-p_{N}\right)}F_{p_{N},\left(n_{N}-p_{N}\right)};T_{N}^{2}\;\epsilon \left[T_{L}^{2},T_{U}^{2}\right]. \end{array}$$

The neutrosophic Hotelling *T*_*N*_^2^ statistic can be used for the testing of hypothesis *H*_0*N*_ : *μ*_*N*_=*μ*_0*N*_ and alternative hypothesis *H*_0*N*_ : *μ*_*N*_ ≠ *μ*_0*N*_. The *H*_0*N*_ : *μ*_*N*_=*μ*_0*N*_ will be rejected if

(17) $$\begin{array}{c} T_{N}^{2}\in \left[T_{L}^{2},T_{U}^{2}\right]>\frac{\left(n_{N}-1\right)}{\left(n_{N}-p_{N}\right)}F_{p_{N},\left(n_{N}-p_{N}\right)}. \end{array}$$

The software provides the *p* value in making a decision about the acceptance or the rejection of the null hypothesis. According to [18], “a neutrosophic *p* value is defined in the same way as in classical statistics: the smallest level of significance at which a null hypothesis *H*_0_ can be rejected.” Note here that the neutrosophic *p* value is not an exact or determined value as in the case of classical statistics. Smarandache [18] discussed criteria to accept or reject the null hypothesis using the neutrosophic *p* value.

## 4. Application

Now, we discuss the application of the proposed neutrosophic Hotelling *T*_*N*_^2^ statistic using data selected from the healthcare department. The data are collected from 20 healthy women and three variables, which are sweat rate, sodium, and potassium contents are measured. The observations of variables underinvestigated will be obtained from the measurement process. It is expected that not all observations in the data are precise and exact. Therefore, it cannot be analyzed using CS. Similar data for classical statistics are given by [28]. The data having some neutrosophic observations are shown in Table 1. We want to test that the means of three groups for the healthy women have the same population means. We state null and alternative hypotheses as follows:

Step 1: $H_{0N}:\mu_{0N}=\left[\begin{array}{c} \left[4,4\right]\\ \left[50,50\right]\\ \left[10,10\right] \end{array}\right]$ vs $H_{1N}:\mu_{1N}\neq \left[\begin{array}{c} \left[4,4\right]\\ \left[50,50\right]\\ \left[10,10\right] \end{array}\right]$.
Step 2: some basic calculations for the data are given in Table 1 are shown as
  (18) $$\begin{array}{c} {\bar{X}}_{N}=\left[\begin{array}{c} \left[4.64,4.70\right]\\ \left[45.5,45.47\right]\\ \left[9.965,10.1\right] \end{array}\right],\\ \mu_{0N}=\left[\begin{array}{c} \left[4,4\right]\\ \left[50,50\right]\\ \left[10,10\right] \end{array}\right],\\ D_{N}=\left({\bar{X}}_{N}-\mu_{N}\right)=\left[\begin{array}{c} \left[0.64,0.70\right]\\ -4.6,-4.53\\ -0.035,0.01 \end{array}\right],\\ S_{N}=\left[\begin{array}{ccc} \left[2.8793,2.8389\right] & \left[10.01,10.04\right] & \left[-1.8090,-1.8284\right]\\ & 199.7884,200.71 & \left[-5.64,-5.72\right]\\ & & \left[3.6276,3.5830\right] \end{array}\right]. \end{array}$$
Step 3: let *α*=[0.10, 0.10] be the level of significance.
Step 4: the neutrosophic Hotelling *T*_*N*_^2^ statistic is
  (19) $$\begin{array}{c} T_{N}^{2}=\left[n_{L}{\left(D_{L}\right)}^{\prime }{\left(S_{L}\right)}^{-1}\left(D_{L}\right),n_{U}{\left(D_{U}\right)}^{\prime }{\left(S_{U}\right)}^{-1}\left(D_{U}\right)\right],\\ T_{N}^{2}=20\left[\begin{array}{ccc} \left[0.64,0.64\right] & \left[-4.6,4.6\right] & \left[-0.035,-0.035\right] \end{array}\right]\\ {\left[\begin{array}{ccc} \left[2.8793,2.8389\right] & \left[10.01,10.04\right] & \left[-1.8090,-1.8284\right]\\ & \left[199.7884,200.71\right] & \left[-5.64,-5.72\right]\\ & & \left[3.6276,3.5830\right] \end{array}\right]}^{\prime }\left[\begin{array}{c} \left[0.64,0.64\right]\\ \left[-4.6,4.6\right]\\ \left[-0.035,-0.035\right] \end{array}\right]=\left[9.7387,11.4176\right]. \end{array}$$
Step 5: the critical region is using equation (5) is given as
  (20) $$\begin{array}{c} T_{0N}^{0}=\frac{\left(20-1\right)}{\left(20-3\right)},\\ F_{0,10,3,17}=\left[8,17,8,17\right]. \end{array}$$
Step 6: as *T*_*N*_^2^=[9.7387, 11.4176] > *T*_0*N*_^0^=[8.17, 8.17], we reject $H_{0N}:\mu_{0N}=\left[\begin{array}{c} \left[4,4\right]\\ \left[50,50\right]\\ \left[10,10\right] \end{array}\right]$.

## 5. Comparisons

In Section 4, we presented the testing procedure for the proposed neutrosophic Hotelling *T*_*N*_^2^. The proposed neutrosophic Hotelling *T*_*N*_^2^ is the generalization of CS. The proposed neutrosophic Hotelling *T*_*N*_^2^ testing procure reduces to the testing procedure under CS when all observations of sweat data are precise. From neutrosophic sweat data, we note that the proposed testing procedure provides the analysis values in the indeterminacy interval rather than the determined values. The neutrosophic form of proposed Hotelling statistic is *T*_*N*_^2^=9.7387 − 11.41*I*_*N*_; *I*_*N*_*ϵ*[0,0.1470]. For example, the proposed Hotelling statistic has the indeterminacy interval from 9.73 to 11.41. It means, under uncertainty environment, one can expect the values of *T*_*N*_^2^ from 9.73 to 11.41. The first value 9.73 of the indeterminacy interval of *T*_*N*_^2^ shows the determined part, and 11.41 is an indeterminate part. When imprecise observations are noted in the sweat data, the value of *T*_*N*_^2^ is 9.73 which is under the CS. In other words, when the level of significance is 5%, the probabilities that the null hypothesis is accepted, rejected, and indeterminate are 0.95, 0.50, and 0.1470. By comparing the proposed test with the test under CS, we note that the existing test is unable to tell about the probability of the indeterminacy. As mentioned by [19, 20] that a method that provides the values in an indeterminacy interval under uncertainty is considered as the most effective and adequate method. By comparing the proposed testing procedure with the existing under CS, our theory is the same as in [19, 20].

## 6. Concluding Remarks

In this paper, we introduced the Hotelling T-squared statistic under neutrosophic statistics (NS) which is the generalization of classical statistics and applied under uncertainty environment. We discussed the application and advantage of neutrosophic Hotelling T-squared statistic with the aid of data. The proposed neutrosophic Hotelling T-squared statistic is expressed in the indeterminacy interval and hence more flexible and information than the Hotelling T-squared statistic under classical statistics. Based on the comparison, we recommend using the proposed neutrosophic Hotelling T-squared statistic for the analysis of the data under uncertainty. Some more properties of the proposed neutrosophic Hotelling T-squared statistic can be studied as future research. The sensitivity of the proposed statistic to uncertainty and measurement errors can be studied in future work.

## Figures and Tables

**Table 1:** The neutrosophic sweat data.

Individual	*X*_1_ (sweat level)	*X*_2_ (sodium)	*X*_3_ (potassium)
1	[3.7, 3.7]	[48.5, 48.7]	[9.3, 9.3]
2	[5.7, 5.8]	[65.1, 65.1]	[8.0, 8.1]
3	[3.8, 3.8]	[47.2, 47.3]	[10.9, 10.9]
4	[3.2, 3.3]	[53.2, 53.3]	[12.0, 12.0]
5	[3.1, 3.1]	[55.5, 55.5]	[9.7, 9.8]
6	[4.6, 4.8]	[36.1, 36.2]	[7.9, 7.9]
7	[2.4, 2.4]	[24.8, 24.8]	[14.0, 14.0]
8	[7.2, 7.3]	[33.1, 33.2]	[7.6, 7.7]
9	[6.7, 6.7]	[47.4, 47.4]	[8.5, 8.6]
10	[5.4, 5.5]	[54.1, 54.2]	[11.3, 11.3]
11	[3.9, 3.9]	[36.9, 36.9]	[12.7, 12.7]
12	[4.5, 4.6]	[58.8, 58.9]	[12.3, 12.4]
13	[3.5, 3.5]	[27.8, 27.9]	[9.8, 9.8]
14	[4.5, 4.5]	[40.2, 40.2]	[8.4, 8.5]
15	[1.5, 1.7]	[13.5, 13.5]	[10.1, 10.2]
16	[8.5, 8.5]	[56.4, 56.5]	[7.1, 7.1]
17	[4.5, 4.7]	[71.6, 71.9]	[8.2, 8.2]
18	[6.5, 6.5]	[52.8, 52.8]	[10.9, 10.9]
19	[4.1, 4.2]	[44.1, 44.2]	[11.2, 11.3]
20	[5.5,5.5]	[40.9, 40.9]	[9.4, 9.5]

## Data Availability

The data used to support the findings of this study are included in the paper.

## References

[1] Caucci L., Barrett H. H., Devaney N., Rodríguez J. J. (2007). Application of the Hotelling and ideal observers to detection and localization of exoplanets. *Journal of the Optical Society of America A*.

[2] Shabbak A., Midi H. (2012). An improvement of the Hotelling T^2^ statistic in monitoring multivariate quality characteristics. *Mathematical Problems in Engineering*.

[3] Brereton R. G. (2016). Hotelling’s Tsquared distribution, its relationship to the Fdistribution and its use in multivariate space. *Journal of Chemometrics*.

[4] Varmuza K., Filzmoser P. (2016). *Introduction to Multivariate Statistical Analysis in Chemometrics*.

[5] Hervé M. R., Nicolè F., Lê Cao K.-A. (2018). Multivariate analysis of multiple datasets: a practical guide for chemical ecology. *Journal of Chemical Ecology*.

[6] Kitagaki B. T., Pinto M. R., Queiroz A. C., Breitkreitz M. C., Rossi F., Nagao R. (2019). Multivariate statistical analysis of chemical and electrochemical oscillators for an accurate frequency selection. *Physical Chemistry Chemical Physics*.

[7] Mason R. L., Chou Y.-M., Young J. C. (2001). Applying Hotelling’s T^2^ statistic to batch processes. *Journal of Quality Technology*.

[8] Zhao N., Zhan X., Guthrie K. A., Mitchell C. M., Larson J. (2018). Generalized Hotelling’s test for paired compositional data with application to human microbiome studies. *Genetic Epidemiology*.

[9] Taleb H., Limam M., Hirota K. (2006). Multivariate fuzzy multinomial control charts. *Quality Technology & Quantitative Management*.

[10] D’Urso P. (2017). Exploratory multivariate analysis for empirical information affected by uncertainty and modeled in a fuzzy manner: a review. *Granular Computing*.

[11] Bakdi A., Kouadri A. (2017). A new adaptive PCA based thresholding scheme for fault detection in complex systems. *Chemometrics and Intelligent Laboratory Systems*.

[12] Ammiche M., Kouadri A., Bakdi A. (2018). A combined monitoring scheme with fuzzy logic filter for plant-wide Tennessee Eastman Process fault detection. *Chemical Engineering Science*.

[13] Azadeh A., Ghaderi S. F., Pashapour S., Keramati A., Malek M. R., Esmizadeh M. (2017). A unique fuzzy multivariate modeling approach for performance optimization of maintenance workshops with cognitive factors. *The International Journal of Advanced Manufacturing Technology*.

[14] Sunanta O. (2018). Generalized point estimators for fuzzy multivariate data. *Austrian Journal of Statistics*.

[15] Yoo C. K., Vanrolleghem P. A., Lee I.-B. (2003). Nonlinear modeling and adaptive monitoring with fuzzy and multivariate statistical methods in biological wastewater treatment plants. *Journal of Biotechnology*.

[16] Smarandache F. (2010). Neutrosophic logic-A generalization of the intuitionistic fuzzy logic. *Multispace & Multistructure. Neutrosophic Transdisciplinarity (100 Collected Papers of Science)*.

[17] Aslam M. (2018). A new sampling plan using neutrosophic process loss consideration. *Symmetry*.

[18] Smarandache F. (2014). Introduction to neutrosophic statistics. *Infinite Study*.

[19] Chen J., Ye J., Du S. (2017). Scale effect and anisotropy analyzed for neutrosophic numbers of rock joint roughness coefficient based on neutrosophic statistics. *Symmetry*.

[20] Chen J., Ye J., Du S., Yong R. (2017). Expressions of rock joint roughness coefficient using neutrosophic interval statistical numbers. *Symmetry*.

[21] Aslam M. (2018). Design of sampling plan for exponential distribution under neutrosophic statistical interval method. *IEEE Access*.

[22] Aslam M. (2019). Product acceptance determination with measurement error using the neutrosophic statistics. *Advances in Fuzzy Systems*.

[23] Aslam M. (2019). Attribute control chart using the repetitive sampling under neutrosophic system. *IEEE Access*.

[24] Aslam M., Khan N., Khan M. (2018). Monitoring the variability in the process using neutrosophic statistical interval method. *Symmetry*.

[25] Aslam M., Albassam M. (2019). Application of neutrosophic logic to evaluate correlation between prostate cancer mortality and dietary fat assumption. *Symmetry*.

[26] Abdel-Basset M., Gunasekaran M., Mohamed M., Smarandache F. (2019). A novel method for solving the fully neutrosophic linear programming problems. *Neural Computing and Applications*.

[27] Abdel-Basset M., Manogaran G., Mohamed M., Chilamkurti N. (2018). Three-way decisions based on neutrosophic sets and AHP-QFD framework for supplier selection problem. *Future Generation Computer Systems*.

[28] Johnson R. A., Wichern D. W. (2002). *Applied Multivariate Statistical Analysis*.

